# A critical analysis of purchasing arrangements in Kenya: the case of micro health insurance

**DOI:** 10.1186/s12913-018-3863-6

**Published:** 2019-01-18

**Authors:** Kenneth Munge, Stephen Mulupi, Edwine Barasa, Jane Chuma

**Affiliations:** 1 Institution: Health Economics Research Unit, KEMRI Wellcome Trust Research Programme, PO Box 43640 00100, Nairobi, Kenya; 2 Nuffield Department of Medicine, Oxford University, Oxford, UK; 3 The World Bank, Kenya Country Office, Nairobi, Kenya

**Keywords:** Micro Insurance, Micro health Insurance (MHI), Strategic purchasing, Low- and middle-income countries (LMIC), Health financing, Universal health coverage

## Abstract

**Background:**

Strategic purchasing can ensure that financial resources are used in a way that optimally enhances the attainment of health system goals. A number of low- and middle-income countries, including Kenya, have experimented with micro health insurance (MHIs) as a means to purchase health services for the informal sector. This study aimed to examine the purchasing practices of MHIs in Kenya.

**Methods:**

The study was guided by an analytical framework that compared purchasing practices of MHIs with the ideal actions for strategic purchasing along three pairs of principal-agent relationships (government-purchaser, purchaser-provider and citizen-purchaser). The study adopted a qualitative descriptive case study design with 2 MHIs as cases. Data were collected through document reviews (regulation, marketing materials, websites) and semi-structured interviews with key informants (*n* = 27).

**Results:**

The regulatory framework for MHIs did not adequately support strategic purchasing practice and was exacerbated by poor coordination between health and financial sectors. The MHIs strategically contracted health providers over whom they could exercise bargaining power, sometimes at the expense of quality. There were no clear channels for beneficiaries to provide timely feedback to the purchaser. MHIs premium payments were family-based, low-cost and offered limited benefits. Coverage was based on ability to pay, which may have excluded low-income households from membership.

**Conclusions:**

Adequate policy, legal and regulatory frameworks that integrate MHIs into the broader health financing system and support strategic purchasing practices are required. The state departments responsible for finance and health should form coordinating structures that ensure that MHI’s role in universal health coverage is owned across all relevant sectors, and that actors, such as regulators, perform in a coordinated manner. The frameworks should also seek to align purchasers’ relationships with providers so that clear and consistent signals are received by providers from all purchasing mechanisms present within the health system.

## Background

Universal health coverage (UHC) is a global agenda with particular relevance for low- and middle-income countries (LMICs), whose populations face challenges in access to services of sufficient quality when needed, and remain at risk of health related impoverishment [1]. For countries to make progress to UHC, it is important that their health financing systems promote UHC goals: revenue generation mechanisms to which contributions are fair and offer financial risk protection [2]; pooling arrangements that reduce fragmentation and allow for effective income and risk cross subsidization to ensure equity and sustainability [3]; and purchasing arrangements that actively pursue the best possible ways to optimize quality, efficiency, equity and responsiveness of health service provision [4].

Purchasing involves determining what to buy (benefit package), for whom (target beneficiaries), from whom (healthcare providers) and for what price [4]. Purchasing can be passive or active also known as strategic purchasing [1]. The former implies a reliance on historical patterns of priority setting, resource allocation and financial management while strategic purchasing involves seeking the optimal way to organize these activities with the aim of reaching health system goals [5]. Strategic purchasing, therefore, involves performing the purchasing activities in a way that continuously seeks to promote quality, efficiency, equity and responsiveness of the health system [4, 5]. Strategic purchasing is critical for the attainment of UHC [6], and is arguably even more important for micro insurance, which targets low-income earners, who often have higher need for health services.

Micro insurance refers to insurance services targeting principally low-income earners, who are excluded from mainstream commercial and social insurance schemes, due to affordability barriers [7–11]. Generally, micro insurers provide a limited set of benefits to members at low premiums, making them affordable to low-income households.

Micro health insurance (MHI and also referred to as HMI) refers to the provision of health insurance services to these households in exchange for premiums charged on the basis of the risk involved [8, 12]. MHI can improve access to health services, offer financial risk protection through reduction in out of pocket expenditures [12]. It has been shown that MHIs, and micro insurance firms in general, tend to have small risk pools and face difficulties in generating sufficient resources [7] which can impact their ability to act as strategic purchasers. More specific evidence on purchasing arrangements suggests that MHI face challenges in designing benefit packages that remain affordable while offering sufficient coverage, obtaining health service providers who offer quality and efficiency, and managing claims in an administratively efficient way [12].

Some LMICs have experimented with micro health insurance (MHIs) to purchase health services [7, 13]. It is thought that in these settings MHI can expand social protection mechanisms; reach groups usually excluded from health insurance such as the informal sector; address gender-inequalities in access to insurance; be designed to better suit customer preferences; and offer better accountability arrangements for financial and service delivery performance [8]. The limited empirical evidence shows that strategic purchasing practice is not widespread among MHI [14–17]. The key aspect of strategic purchasing employed was determining who to buy from with evidence of selection and contracting based on capacity, quality and cost considerations [13, 15, 17]. Other activities identified were benefit package design, where the trade-off between affordability and comprehensiveness was identified; and the use of provider payment mechanisms with fee for service and budgets being the most widely used forms [13, 15, 17].

### Health micro insurance in Kenya

As with other LMICs, Kenya’s pursuit of UHC has gained momentum and several reforms have been implemented. These include the removal of user fees in government-owned dispensaries and health centres and the introduction of free maternity services in public facilities [18, 19]. Other reforms have targeted the National Hospital Insurance Fund (NHIF), Kenya’s social health insurance scheme, and include expansion of the benefit package to include outpatient and specialized services such as renal dialysis, an increase in contribution rates, and governance restructuring [20–22]. Despite these efforts, only 17% of Kenyans had any form of health insurance in 2013 [23], and this number is unlikely to have increased significantly in the recent past. Health insurance coverage remains low especially among the poor and low-income households who mainly work in the informal sector, which makes up 83% of Kenya’s labour force [23–26]. For example, provisional estimates from 2015 show that only 16% of all informal sector workers in Kenya have NHIF insurance [27]. The low levels of insurance coverage present a challenge for Kenya at a time when mandatory health insurance is being considered as a main financing mechanism for UHC [28–30]. Micro insurance is expected to play a role in the country’s progress towards UHC and broader social protection [28, 31].

Micro-insurance schemes are categorized in several models: partner-agent, provider-driven, mutual model (community-based) and the direct agent model [7, 8, 32]. In the partner-agent model, an insurance firm contracts another entity to sell it products, while under direct agent model, an insurance firm controls the entire chain and sells its product directly to consumers [8]. The mutual or community-based model is owned and run by scheme members through participatory mechanisms [7, 8, 33]. The provider driven model is led by a health service provider who takes on an insurance function [7]. All four models of micro insurance are present in the Kenyan health insurance context. The provider-driven model and the community-based model are frequently lumped together and referred to as community-based health insurance (CBHI) schemes [34]. These two models lack a legal and policy framework and tend to be administered as societies or community-based organisations [33, 35]. On the other hand, the partner-agent and direct agent models are labelled as MHIs and have received increasing policy and regulatory attention from the Kenyan government [31, 36].

There are other features that distinguish CBHI and MHIs in Kenya. CBHI operate within a specific small geographical location [33, 37], while MHIs operate nationally [38]. Access to services for CBHI members is usually restricted to low-cost public facilities and faith based facilities within the same geographical area, while MHIs have a nationwide network of low- to mid-cost public and private providers [35]. Additionally, while MHIs offer risk rated premiums to members calculated individually or for groups, CBHI schemes base premiums on members’ decisions, informed by very limited financial analysis, if any [33].

There is no accurate data on the number of and population covered by MHIs in Kenya, largely due to weak regulation, and existing reporting requirements where their activities are recorded under the insurance firms that underwrite the products [31, 38]. A report published in early 2014 suggested that about 300,000 lives were covered: about 1% of Kenya’s population [39].

There is growing interest in Kenya and globally on the role of MHIs as a mechanism for health financing towards UHC [7]. MHI are not only of interest to health policy makers, but also to those from the finance and social sectors who may be interested in the financial and social inclusion potential of MHI [8, 40]. This concern has not been matched with a systematic examination of whether these purchasing practice of MHI are strategic. This paper addresses this evidence gap by examining purchasing practices of MHIs and the extent to which these are aligned to strategic purchasing.

## Methods

### Study setting

Kenya’s health system is organized around two major administrative levels: the national, that is primarily responsible for policy, regulation and national referral facilities; and the county level that is largely responsible for service delivery. Health care services are provided by both private (for-profit and not-for-profit) and public providers in almost equal shares [41]. Health care services, especially in the public sector, are arranged in tiers from community through to tertiary care [42]. Health is financed by the government (31% of total health expenditure, THE), private sources (mainly through out of pocket payments which are 32% of THE) and donors (25% of THE) [43]. The majority of Kenya’s insured population (88%) are covered by the NHIF [23]. The rest are covered by private health insurance which includes MHIs (9%), employer-based medical schemes (about 3%) and CBHIs (< 1%) [23]. At the time of this study, there were 18 private health insurers and 29 medical insurance providers in Kenya; the latter being a special class of health insurance providers who do not underwrite their own products [36, 44].

### Study design and data collection

The study adopted a qualitative descriptive case study design. Two MHIs were purposively selected as case studies based on discussions with policy makers. The first organization (MHI Case A) was a mobile phone innovation focussed firm, which had partnered with a telecommunications company and an insurance underwriter to develop and market a health micro insurance product. The second (MHI Case B) was a leading micro finance institution that had partnered with the same insurance underwriter. The insurance underwriter (Private Insurance Firm C) was one of the largest insurance firms in Kenya and also provided micro health insurance under a direct agent model. Though both were partner-agent models, there was the view that they would provide sufficient data to meet the objective of the study and that any gaps would then be filled by the experiences of Private Insurance Firm C. The characteristics of the cases are summarized in Table 1:

**Table 1:** Summary of Case Study Micro Health Insurance Firms

	MHI Case A	MHI Case B
Model	Partner-agent model	Partner-agent model
Underwriter		Private Insurance Firm C
Area of operation	National	National
Benefit package [45, 46]	Outpatient and inpatient services Family-based cover Other benefits: Daily hospital cash, Funeral benefit, Cover limit in two bands KES 139,000 and KES 290,000 No premium financing	Outpatient and inpatient services Family-based cover Other benefits: Life insurance cover, Funeral benefit, Cover limits in three bands KES 200,000, KES 500,000 and KES 1,000,000 Premium-financing available through micro-finance loan
Premium rate [45, 46]	KES 12,000 for full benefit	Not determined
Number of members	About 8000 in late 2014^a^ [46]	About 12,000 in 2014 [39]
Health service providers	Public and low- and mid-cost private health facilities	Public and low- and mid-cost private health facilities

^a^as many as 65,000 at fold up in 2015 [47]

Data were collected between April 2014 and May 2015, through document review and semi-structured interviews with key informants. Documents reviewed included statutes, reports, policy documents, websites content and grey literature. Specific to the two MHIs, we reviewed websites, sales material documenting their benefit packages, and project evaluation reports. Documents were retrieved by the authors independently and also obtained from respondents. Data were extracted into standardised forms and a summary assessment of the reviewed documents was produced. Semi-structured interviews were conducted with micro health insurance providers (1 from Case A and 1 from Case B), micro health insurance underwriters (1 underwriting both Case A and B, and 2 underwriting other MHI) and regulators (2 regulating both Case A and B). Additional interviews were conducted with independent actuaries (*n* = 2), other private health insurance players (*n* = 8), industry lobby groups (*n* = 1) and health service providers (*n* = 9). Table 2 presents a summary of the respondents.

**Table 2:** Summary of respondents

	Number interviewed		
Respondent	MHI Case A	MHI Case B	Other
MHI providers	1	1	
MHI underwriters	1		2
Insurance regulators			2
Health Service Providers			9
Other private insurance firms			8
Independent actuaries and consultants			2
Insurance industry association/lobby group			1

Respondents were approached through phone and email, informed of the general objectives of the study and requested to participate in an interview. Semi-structured interviews were conducted face-to-face at the respondents’ place of work. Written informed consent was sought before the commencement of each interview. Interview questions were based on the conceptual framework (Table 3). Interviews took about 1 h and were audio recorded, with field notes taken by the accompanying researcher who also sought clarification on key points during the interview.

**Table 3:** Strategic Purchasing actions

Government-Purchaser axis	
Governments should provide policy and regulatory frameworks that support strategic purchasing	
Purchaser-Provider axis	
Purchasers should select and contract health service providers based on criteria including capacity and geographical distribution	
Purchasers should require providers to ensure quality of service including through quality improvement mechanisms such as the use of standard treatment guidelines	
Purchasers should incentivize provider performance through payment mechanisms and related incentives	
Citizen-Purchaser axis	
Benefit packages should reflect needs and preferences of the target population	
Benefit packages should offer protection from financial catastrophe	

Source: Adapted from RESYST [48]

### Conceptual framework

The study was guided by an analytical framework summarized in Table 3. The purchasing practices of MHIs were compared with the ideal actions for strategic purchasing along three pairs of principal-agent relationships (government-purchaser, purchaser-provider and citizen-purchaser) proposed by the Responsive and Resilient Health Systems (RESYST) Consortium [48]. Drawing on agency theory and guided by Figueras et al’s framework [4], the RESYST framework specifies actions that the purchaser can take to ensure that the agent acts in a way that benefits the principal. The description of ideal actions is based on reviews of the existing literature on strategic purchasing and the experiences of the reviewers and consortium members who are experts in health financing. For this study, the government-purchaser axis was approached from an industry-wide perspective, with the government including the national government and insurance industry regulators in charge of the micro insurance sector as a whole. The purchaser-provider and provider-citizen axes were approached from a scheme specific perspective using the two MHIs as cases.

### Data analysis

The audio recordings were transferred onto a secure computer, assigned a unique code and transcribed verbatim before being checked for consistency and correctness. Checked transcripts were then transferred into QSR NVivo 10 for coding and analysis. Coding was performed using thematic framework analysis with themes drawn from the analytical framework. Two researchers (KM and SM) coded the transcripts separately. Contentious coding outputs were agreed through consensus with a senior researcher (EB and JC). A feedback meeting was held with respondents to obtain their views of the study results. Other means of ensuring rigor of the study included the triangulation of interview data with data drawn from documents, and the utilisation of more than one organization to provide as wide a range of data as possible.

## Results

### Government-purchaser relationship

#### Inadequate regulatory framework

Kenya lacks a specific health insurance law and regulatory framework. Health insurance is governed by the Insurance Act [36], and registration is done through the Insurance Regulation Authority (IRA). However, the Insurance Act does not cover key aspects of health insurance and the IRA lacks adequate capacity to handle aspects related to health insurance. Specifically, there are no guidelines on contribution rates and corresponding or minimum benefit package, nor are there any general indications for selecting providers. This inadequacy extends to micro insurance with few health-specific provisions in insurance policy and regulatory frameworks. For example, the Micro Insurance Policy Paper proposes that waiting periods for maternity, surgical and other benefits be 9 months, 6 months and 2 months respectively [31]. These proposals are not reflected in existing insurance law or in draft regulations and there are currently no guidelines supporting their implementation [36, 49]. They are also not linked to any particular health policy or regulation and may therefore not address health goals of increasing access to care [50].

### Purchaser-provider relationship

#### Strategic provider selection

The MHIs preferentially contracted low- and mid-tier cost public and private health providers over whom they could exercise bargaining power by offering their client base to them. Neither of the MHIs was involved in the selection and contracting process; they instead leveraged on the experience and capacity of the underwriter, Firm C. Nevertheless, the MHIs communicated client preferences for health service providers to Firm C.

*“So whenever we are contracting you the first thing we want to know [is] these kind of services how much do you charge…so if the answer is that it’s more than X which is our average, we don’t want to work with you. If your answer is X and below then, we’re happy to work with you simple.” PHI_10*

#### Weak quality assurance

Focusing on cost of care sometimes undermined quality of services. The challenge to attaining good quality of services was clear to the MHIs manifested for example as long waiting times.

*“The challenge is there is always the view that…these people will not pay immediately especially if there are those who are paying cash. And they will not…get…fast service because maybe when you come with the card, there is a process you must follow…” PHI_10*

In order to address quality assurance, the MHIs piggybacked on measures undertaken by Firm C where these existed. From document review and interviews, these included accreditation, regular inspection and customer satisfaction reviews. However, these measures were variably applied owing to a lack of capacity and a poorly applied overarching quality framework for the health sector. Key quality improvement levers such as clinical guidance and support mechanisms or monitoring and performance measurement were not in use or were left to providers’ professional judgement and discretion.

Beneficiaries who experienced poor quality services were unable to present timely feedback owing to a lack of clear communication channels between themselves and the purchaser. This was contributed to in part by the existence of multiple intermediaries in the chain between the beneficiary and the purchaser. For example, in Case B had developed a call centre that filtered calls before onward submission to the insurance underwriter, Firm C. However, Case B had no means of ensuring quality issues were taken care of, as this was legally the responsibility of the underwriter.

*“Well because we have this call centre we are then able to prioritize…then decide who is responsible and [Private Insurance Firm C] have a team that actually engages with providers or customer complaints.” PHI_01*

#### Weak incentives for provider performance

The provider payment mechanism utilised by both MHIs was fee-for-service, linked to their use of the same insurance underwriter. In keeping with theory and evidence, there was the perception that this might have resulted in overuse and fraud.

*“…. by and large if you look at the health insurance particularly from the outpatient point of view, the abuses and the fraud that underlies it is enormous.” KII_18_industry association*

Providers resisted movement to new forms of provider payment because of concerns over the way in which rates were estimated, and providers had power over purchasers. This power was drawn in part from their limited supply and their control over key rate setting mechanisms [51].

*“So, the resistance simply comes as, ‘We don’t want to take capitation and we know you are still going to use our services.’ If they knew that they will miss out on the business, if they didn’t take capitation then the behavior of many of these providers will change…” PHI_06*

*“Capitation has been attempted a few times…but it has really not picked up properly…there is need for a proper actuarial study to be commissioned where there is no vested interest, because a lot of times somebody say we are doing an actuarial study but they have got vested interests, so the result is already skewed to favour what you want to come out of it.” KII_23_provider*

### Purchaser – Citizen relationship

#### Coverage based on ability to pay

MHIs premium payments were family-based and could be characterized as “low-cost, low-cover”. However, coverage and therefore access to services was based on ability to pay. For example, Case A offered three levels of financial limits depending on the premium paid. Similarly, Case B offered restricted access to benefits to those who paid half of the annual per household premium (KES 6000; USD 60) and full access to those paid the annual premium (KES 12,000; USD 120).

This may have restricted access to the poor and informal sector, which is characterized by low and irregular income. Case A addressed this problem by offering loans through its microfinance platform to finance premiums.

*“The option of loan…we let them be in groups where they can guarantee one another and repay every week a small premium to cater for their medical; so that we pay full premium for them and then they slowly by slowly repay the loan” PHI_11*

Case B encouraged users to save up to the premium and used its partnership with a telecommunications company to encourage mobile-based payments. However, the effectiveness of these solutions is questionable given the low uptake of products offered by both MHIs. For example, Case B has since withdrawn its product and private insurance Firm C now only offers products that charge higher premium rates that are restricted to groups of minimum 10 members.

#### Inadequate financial risk protection

Another barrier to financial risk protection was the absence of caps on balance billing, which meant that providers could charge users directly for costs that exceeded their cover limit. These top-up payments would therefore need to be made out-of-pocket exposing households to financial catastrophe. However, this was mitigated somewhat by the deliberate contracting of low- and middle-cost health service providers, and with integrating the packages with NHIF cover, which covered some of the cost of care.

*“When you design a product, a key component into the product benefits and the pricing, will be the type of provider you use. For a faith-based hospital or a commercial high-end hospital the pricing will be different.” PHI_04*

#### Needs-based benefit package design

MHIs partnered with the insurance underwriter to assess health needs through market analysis, customer surveys and feedback, mystery shopping and intelligence gathering from competitors. For example, Case A used internal intelligence gathered as a microfinance institution to influence product design and distribution. Their data suggested that potential users preferred easy to understand products as well as continuous engagement. As a result, both MHIs benefit packages were simple, provided unique features such as funeral expense benefit, and had innovative options for premium payments, for example, through the use of mobile phones. Waiting periods for services, such as maternity services, were shorter than conventional insurance.

*“When the customer comes in for a loan, we also tell him ‘You see we have another product here which will be able to assist your family.’” PHI_11*

These needs assessment activities also served the insurer well by reducing complaints and improving the chances for dispute resolution.

*“When people know what they’re buying, they will not complain because our message always is…‘We want to tell you what we’re selling to you’…” PHI_10*

However, benefit packages still excluded important preventive health services such as immunizations and vaccines, annual health checks, and family planning. Moreover, the use of evidence did not extend to more robust sources with cost cited as an impediment.

*“…Research is a very expensive venture, we try to do as often as we can but of course because of the cost again it’s not possible.” PHI_10*

## Discussion

We have presented here how health micro insurance in Kenya functions in terms of strategic purchasing practice. The shortcomings are identifiable in each of the four decisions purchasers must make, i.e. what to buy, for whom, from whom and how.

Overall, the shortcomings in the four decision making domains were due to an inadequate policy and regulatory framework for strategic purchasing. This concern is echoed by previous work in Kenya and other LMIC [35, 52–54]. This is a more significant problem for MHIs, which operate under a variety of labels, mandates, motivations and even sectoral contexts. In the case of Kenya, the recent micro insurance policy [31] and amendments to the Insurance Act [36] represent missed opportunities for embedding strategic purchasing practice in a nascent form of insurance. This may be attributed to lack of stewardship by the government and limited stakeholder engagement [55]. In addition, the Insurance Regulatory Authority (IRA), under whose remit MHIs and insurance regulation fall, is answerable to the Ministry of Finance, with limited engagement, if any of the Ministry of Health (MOH). This arrangement potentially contributed to weak policy coordination between the IRA and the MOH. The intersection of financial and health sectors presents an opportunity for collaboration and coordination that would allow MHIs to contribute to health financing arrangements [56].

The inadequacy of policy and regulatory frameworks may result in unintended outcomes. For example, we identified the use of loans for premium financing through microfinance mechanisms. While this may be viewed as a means of addressing affordability and access to the package of health services, there is evidence that microfinance loans can have adverse effects if poorly implemented. In particular, evidence from India highlights that poorly designed and implemented microfinance solutions can contribute to financial strain in households [57, 58]. Though these experiences have not been documented in Kenya, they provide relevant policy lessons related to premium rates, a concern relevant to strategic purchasing. Where premium rates increase over time they may exert increasing pressure on households.

In the absence of overarching guidance on what to buy and for whom, MHIs showed some ability to reflect member needs and preferences in the design of benefit packages, based on feedback from providers and beneficiaries (informal sector workers and other low-income earners). However, the extent to which this information was used to revise the benefit package was limited to keeping the package succinct and simple. There was little use of robust sources of evidence to inform service entitlements with the result that some cost-effective services were excluded from benefit packages. In addition, the focus on curative care failed to address the changing pattern of disease in Kenya, which may require a greater focus on health promotion and prevention. For example, non-communicable diseases, which are potentially preventable and require health promotion, contributed 30% (95% CI 26.4–33.3) of the total DALYs and 27% (23.4–31.5) of all deaths in Kenya in 2016 [59]. A review of CBHI benefit packages offered in sub-Saharan Africa in 2011 revealed that most offered limited benefits with high co-payments [13].

Provider selection, based on capacity to deliver services and to expand geographical access, is essential to ensuring that beneficiaries can access their entitlements [4]. MHIs demonstrated strengths in selecting providers who fulfilled these requirements. However, shortages in supply of health services can reverse the power relationship between purchasers and providers and therefore limit strategic purchasing practices that depend on the bargaining power of the purchaser. Evidence from Indonesia, though not drawn from micro insurance, suggests that ensuring quality remains a challenge in settings where providers are limited in supply [60]. This finding is supported by work in Kenya which showed that purchasers were sometimes forced to accept providers’ prices [35]. This evidence suggests that MHIs would face similar limitations even with an expressed intent to select appropriate providers. The main solution to this problem lies with the government, which stewards the health system. Several options exist including steps to train, attract and retain health workers in underserved areas [61]; and to discourage the over-supply of services in certain geographical areas [62]. MHIs could also influence the pattern of distribution by leveraging on member location to influence provider location and service type.

In terms of how to purchase, MHIs had a limited suite of incentives and sanctions over providers, which meant that they could not guarantee the quality or efficiency of services provided. This problem is observable in other purchasing mechanisms in Kenya and other LMIC [35, 52, 63]. For example, the use of contracts may be limited by the absence of a law or compulsion to contract certain types of providers. Diverse forms of provider payment mechanisms may also require appropriate regulation or financial management requirements. The application of sanctions may also be constrained by the need to maintain good relations with providers in the face of provider market power, as discussed above. More specifically, evidence from India suggests that MHIs have limited influence over providers especially where they continue to lie outside broader government initiatives to improve service availability and quality [64]. MHIs can take steps to ensure their suite of incentives and sanctions align with broader health system arrangements and goals. However, government can also undertake to provide frameworks for strategic purchasing that support system-wide approaches to introducing new provider payment mechanisms [65], other incentives and sanctions, and contractual relationships between purchasers and providers [66].

This study had several limitations. The case studies selected omitted the direct agent model which is also present in Kenya. This limitation is mitigated by the inclusion of Private Insurance Firm C which has experience in implementing the direct agent model. Also, while this paper does not report on the provider-driven model and the community-based models, commonly referred to as community-based health insurance in Kenya, the results of this examination are published elsewhere [35]. The other limitation is the inability to statistically generalize the findings to the rest of the country or other settings. The goal of qualitative analysis is to achieve analytical generalizability (i.e. generalizing to theory) rather than statistical generalisability. The study provides important insights into strategic purchasing practices of MHI that can be considered in the wide range of settings to which the concept of MHI is being applied.

## Conclusions

MHI’s potential role in purchasing health services for low-income earners in Kenya may not make a meaningful impact on the attainment of UHC goals because of the absence of strategic purchasing practices. Adequate policy, legal and regulatory frameworks are required that support strategic purchasing practice in Kenya. Specific to MHI, the frameworks should support the integration of MHIs into the broader health financing system in a way that would enhance progress towards UHC.

Development and implementation of these frameworks will require coordination across sectors. Specifically, state departments responsible for finance and health should form coordinating structures that ensure that MHI’s role in UHC should be owned as an agenda across all relevant sectors, and that actors, such as regulators, perform in a coordinated manner.

This coordinated approach is particularly important in dealing with health service providers in order to optimise efficiency and quality health services. Specifically, the frameworks should seek to align provider selection, performance monitoring, incentives and sanctions, so that clear and consistent signals are received by providers from all purchasing mechanisms present within the health system.

## Abbreviations

CBHI: Community-based health insurance
IRA: Insurance Regulatory Authority
KES: Kenya Shillings
LMIC: Low- and middle-income countries
MHI: Micro Health Insurance
MOH: Ministry of Health
NHIF: National Hospital Insurance Fund
RESYST: Resilient and Responsive Health Systems
THE: Total Health Expenditure
UHC: Universal Health Coverage
